# Consequences of double virus infections on host plant preference and performance of *Aphis gossypii* (Hemiptera: Aphididae)

**DOI:** 10.1093/aesa/saag009

**Published:** 2026-04-16

**Authors:** Rocio Galan-Cubero, Javier Cazorla, Alberto Fereres, Aranzazu Moreno

**Affiliations:** Plant Protection Department, Instituto de Ciencias Agrarias—Consejo Superior de Investigaciones Científicas (ICA-CSIC), Madrid, Spain; Escuela Técnica Superior de Ingeniería Agronómica, Alimentaria y de Biosistemas (ETSIAAB), Universidad Politécnica de Madrid (UPM), Madrid, Spain; Plant Protection Department, Instituto de Ciencias Agrarias—Consejo Superior de Investigaciones Científicas (ICA-CSIC), Madrid, Spain; Plant Protection Department, Instituto de Ciencias Agrarias—Consejo Superior de Investigaciones Científicas (ICA-CSIC), Madrid, Spain; Plant Protection Department, Instituto de Ciencias Agrarias—Consejo Superior de Investigaciones Científicas (ICA-CSIC), Madrid, Spain

**Keywords:** aphid, noncirculative virus, circulative virus, life-history traits, host preference

## Abstract

Understanding virus–vector–host interactions in the context of mixed infections is crucial to predict virus spread and develop effective control measures in economically important crops. The virus transmission process plays a central role in plant disease epidemiology and is particularly influenced by vector behavior and life-history traits. However, how mixed virus infections shape vector fitness and host preference remains poorly understood. Here, we determined the effects of double infections with the noncirculative cucumber mosaic virus (CMV, *Cucumovirus*) and the circulative cucurbit aphid-borne yellows virus (CABYV, *Polerovirus*) on the fitness and host plant preference of the aphid *Aphis gossypii* Glover on the host plant, melon (*Cucumis melo* L.). We revealed that aphids feeding on melon infected only with CMV (single-infected) weighed less and had lower fecundity than those feeding on uninfected plants or CABYV single- or double-infected plants. The mean relative growth rate and, consequently, the intrinsic rate of natural increase were also lower in aphids on CMV single-infected plants, indicating a negative impact of CMV single-infection on aphid fitness. In choice experiments, aphids were initially attracted to CABYV/CMV double-infected plants, but this was followed by a shift in host preference toward mock-inoculated plants over time. These findings suggest that mixed infections can modulate vector behavior in complex and dynamic ways. The pattern of early attraction followed by dispersal appears to promote CMV spread while having limited consequences for CABYV transmission, indicating an asymmetrical benefit between these 2 viruses.

## Introduction

Virus spread is a crucial element of disease epidemiology, and it is heavily shaped by the behavior and life-history traits of the vectors that carry and transmit these viruses (Cunniffe et al. 2021). Although viruses can be transmitted in many ways, such as by direct contact or via pollen and seeds, the majority are transmitted by insect vectors (Fereres and Raccah 2015). Among these vectors, hemipterans, and more precisely aphids and whiteflies, are responsible for transmitting approximately 50% of all insect-transmitted plant viruses (Brunt et al. 1996, Nault 1997, Bragard et al. 2013). To increase transmission and spread, viruses often trigger physical and chemical alterations in their vector and/or host plant, subsequently influencing interactions between the vector and host (Jiménez-Martínez et al. 2004a, Belliure et al. 2005, Mauck et al. 2010, 2012, Carmo-Sousa et al. 2014, Su et al. 2015, Carmo-Sousa et al. 2016, Domingo-Calap et al. 2020, Chesnais et al. 2021). As stated in the “Vector Manipulation Hypothesis,” pathogens can influence their vectors either indirectly, by inducing modifications in the host plant in response to virus infection, or directly, through virus presence within the vector (Ingwell et al. 2012). These changes stem from coevolutionary adaptation among the pathogen, plant, and vector and appear to be related to virus transmission mode. However, several studies demonstrate that vector manipulation is not universal and that viruses cannot be optimally adapted to manipulate all hosts (García-Arenal and Fraile 2013, Lefeuvre et al. 2019, Mauck and Chesnais 2020, Chesnais et al. 2025).

Viruses are characterized by their mode of transmission as either circulative (C-transmitted, or CT) or noncirculative transmitted (NCT) viruses. CT viruses, also referred to as persistently transmitted viruses, penetrate the insect gut barriers, enter the circulatory system, and eventually accumulate in the salivary glands. In contrast, NCT viruses, also referred to as semi- or nonpersistently transmitted viruses, remain attached to the cuticle of the stylets or foregut without crossing host gut barriers (Fereres and Raccah 2015). Consequently, CT viruses have a closer and more specialized relationship with their vectors than NCT viruses (Mauck et al. 2014, Eigenbrode et al. 2018, Moreno and López-Moya 2020, Jayasinghe et al. 2022).

For CT virus transmission to occur, aphids must access and feed from the phloem tissues of infected plants to efficiently acquire and inoculate the virus. Although some exceptions exist, such as pea enation mosaic virus (PEMV, *Enamovirus*), which can be acquired from mesophyll cells (De Zoeten and Skaf 2001), most CT viruses are strictly phloem-limited. Due to this requirement, CT viruses exhibit a highly specific relationship with their vectors, with only a limited number of vector species (associated with the host plant) able to transmit them (Gildow and Gray 1993, Gray and Gildow 2003). Moreover, CT viruses prompt modifications in their host plants, making them more attractive to their virus vectors, thereby promoting virus spread (Mauck et al. 2012). The behavioral and physiological effects of 2 of the most highly relevant genera of CT viruses in agricultural crops, *Luteoviruses* and *Poleroviruses*, on their insect vectors have been reviewed by Bosque-Pérez and Eigenbrode (2011) and Eigenbrode et al. (2018). Carmo-Sousa et al. (2016) also reported that the CT cucurbit aphid-borne yellows virus (CABYV, *Polerovirus*), which belongs to the family Solemoviridae, induced changes not only in the settling and alighting of *Aphis gossypii* but also in its probing behavior, feeding more frequently, and extending the periods of phloem sap ingestion on infected plants than on uninfected ones. These changes in feeding behavior and host plant preference enhanced CT virus transmission by aphids (Scheller and Shukle 1986, Jiménez et al. 2020).

Furthermore, vector fitness can increase when feeding on plants infected with phloem-limited CT viruses (Araya and Foster 1987). This parameter of vector fitness, understood as vector performance in terms of survival, development, and reproduction under different conditions, provides key information on how virus infections affect the biology of the vector and, consequently, its ability to spread the virus to new host plants. One of the main tools to evaluate vector fitness is life tables, used to analyze parameters such as population growth rate, fecundity, longevity, and mortality at different stages of the vector’s life cycle (Pedigo and Zeiss 1996, Chi et al. 2020). An example of enhanced fitness was observed for *Sitobion avenae* and *Rhopalosiphum padi* that produced more offspring on Barley yellow dwarf virus (BYDV) infected wheat than on mock-inoculated wheat (Fereres et al. 1989, Jiménez-Martínez et al. 2004b). In contrast, plant-mediated effects of turnip yellows virus (TuYV, *Polerovirus*) benefited its vector, Myzus *persicae*, in terms of growth rate and host plant preference, preferentially leaving uninfected hosts in favor of turnip mosaic virus (TuMV)-infected plants (Chesnais et al. 2019).

The effects of CT viruses have also been observed in other insect vectors (Jiang et al. 2000, Dietzgen et al. 2016, Zhao et al. 2022). A representative case was observed by Moreno-Delafuente et al. (2013), who reported that the CT virus, tomato yellow leaf curl virus (TYLCV, *Begomovirus*) manipulated probing, feeding, and settling behavior of its vector, *Bemisia tabaci*, thereby increasing virus transmission and spread. Moreover, Wang et al. (2014) observed that the CT viruses, southern rice black-streaked dwarf virus (SRBSDV, *Fijivirus*) and rice ragged stunt virus (*Oryzavirus*), manipulated their planthopper vectors *Sogatella furcifera* and *Nilaparvata lugens* to enhance their own spread. Similar effects were documented with the CT virus, tomato spotted wilt virus (*Orthotospovirus*), on the performance and host plant preference of *Frankliniella occidentalis*, in which the number of offspring was increased on infected plants (Nachappa et al. 2020).

In contrast to CT viruses, NCT viruses are acquired and transmitted during superficial intracellular punctures of the plant (Powell et al. 1995, Martín et al. 1997, Powell 2005, Moreno et al. 2012). Because NCT viruses are attached to the vector’s mouthparts, their interaction with the vector is less specific than for CT viruses. Furthermore, NCT viruses, such as cucumber mosaic virus (CMV, *Cucumovirus*), which belongs to the family *Bromoviridae*, can initially make infected plants more attractive to aphid vectors, but their suitability to feeding after probing diminishes, leading to rapid spread of vectors from infected to uninfected plants (Mauck et al. 2010, 2014, Carmo-Sousa et al. 2014, Rozo-Lopez and Parker 2023). Moreover, this NCT virus provoked indirect effects on aphid feeding behavior, specifically increasing the number of intracellular punctures, known to favor transmission and spread of NCT viruses (Powell et al. 1995, Martín et al. 1997, Carmo-Sousa et al. 2014).

NCT viruses can also impact aphid reproduction: with the NCT virus, TuMV (*Potyvirus*) infection, promoted *M. persicae* reproduction (Casteel et al. 2014), whereas bean yellow mosaic virus (BYMV, *Potyvirus*) reduced *Acyrthosiphon pisum* reproduction (Hodge and Powell 2008). CMV, the NCT virus central to our study system, shows similarly variable outcomes: it reduces *A. gossypii* frequency and density on infected cucurbits (Mauck et al. 2010) but enhances *M. persicae* reproduction on infected pepper (Warnasooriya et al. 2024). However, as these examples show, the effects of NCT viruses on vector preference and fitness vary among pathosystems.

Virus-induced modifications in host plants, and consequently aphid vectors, have been widely studied in single-infection pathosystems. However, our understanding of mixed virus infections, which have proven to be the rule rather than the exception in nature, is still developing. Many viruses exhibit generalist behavior, enabling them to infect multiple hosts, and numerous plant virus vectors are polyphagous, creating frequent opportunities for coinfection in the field (Alexander et al. 2014, Syller 2014, Moreno and López-Moya 2020).

Mixed infections not only complicate virus interactions but also influence vector behavior and performance, which are key factors in virus epidemiology (Moreno and López-Moya 2020). For instance, these infections could modify symptom expression and alter visual and olfactory cues that influence vector preference. For example, tomato coinfection with the CT virus TYLCV (*Geminivirus*) and the NCT virus tomato chlorosis virus (ToCV, *Closterovirus*) led to more severe symptoms at later stages and altered *B. tabaci* preference toward plants infected either with TYLCV only or with TYLCV and ToCV, with visual cues from TYLCV-infected plants driving host selection (Ontiveros et al. 2022). Furthermore, potato plants infected with both NCT potato virus Y (PVY, *Potyvirus*) and CT potato leafroll virus (PLRV, *Polerovirus*) modified *M. persicae* and *Macrosiphum euphorbiae* behavior not only in terms of fecundity, being significantly higher on double-infected plants than on single or uninfected plants; but also in preference of alatae and apterae of *M. persicae* and *M. euphorbiae*, which preferentially settled on double-infected plants compared with single-infected or uninfected plants (Srinivasan and Alvarez 2007). Double infections may also modify the transmission efficiency of individual viruses, as shown by Wintermantel et al. (2008) for ToCV and semipersistent tomato infectious chlorosis virus (TICV, *Closterovirus*) in *Nicotiana benthamiana*, where double infection enhanced TICV transmission while reducing that of ToCV.

Overall, during mixed infections, viruses can interact in different ways, ranging from synergistic to neutral to antagonistic relationships (Sadras et al. 2024), probably impacting not only the host plant but also virus relationships with their vector (Singhal et al. 2021). These interactions highlight the complex dynamics of mixed infections in plant-virus epidemiology.

In the specific case of cucurbits, which are economically important crops worldwide, production can be affected by several viral diseases (Lecoq and Katis 2014, Rabadán et al. 2023). Most of these viruses are mainly transmitted by insect vectors and belong to families such as Geminiviridae, Closteroviridae, Potyviridae, Bromoviridae, and Solemoviridae, inducing either leaf yellowing or mosaic symptoms, regardless of the cultivar, environmental conditions, or viral strain (De Moya-Ruiz et al. 2023). In particular, CABYV and CMV have been reported infecting major cultivated cucurbit species in Spain, with a significant association and relevant prevalence in mixed infections (Alonso-Prados et al. 2003, Rabadán et al. 2023). Our study aimed to deepen our knowledge of how double infections of melon (*Cucumis melo* L., order Cucurbitales, family Cucurbitaceae) plants involving a CT virus (CABYV) and a NCT virus (CMV) affect the fitness and host plant preference of *A. gossypii* because their virus-mediated indirect effects in single infections have already been assessed (Carmo-Sousa et al. 2014, 2016). To investigate the effects of double infections, we compared vector life-history traits in choice experiments using infected and uninfected plants to study how double infection may impact vector population dynamics, host plant detection, and host plant selection. Understanding these effects is crucial to predict the epidemiological consequences of mixed infections and is particularly relevant to managing virus diseases in cucurbits and other economically important crops.

## Materials and Methods

### Aphid Clones, Host Plants, and Virus Strains

The *A. gossypii* colony was established from a single virginoparous female from a population collected in Almeria (Spain) in 1998. The nonviruliferous aphid colony was reared on melon plants (*C. melo* var. Alcazaba), renewed fortnightly, and maintained in a climatic chamber under controlled conditions at a temperature of 23:20 °C day:night and a photoperiod of 16:8 h light:dark.

Before life-history trait assays, nonviruliferous aphids used in the different treatments were synchronized. Nonviruliferous apterous adult aphids (7 to 9 days old) were selected from the colony available at ICA-CSIC and were placed for 24 h in clip cages on leaves of healthy uninfected melon plants. After this period, adults were removed, leaving the neonate nymphs for 6 to 7 days before use in assays, corresponding to the period needed to reach adulthood.

Melon plants (var. Monique and var. Rochet) were used for both the life-history trait and previous host plant preference experiments. All plants were sown in cell trays and transplanted at stage 12 (BBCH scale) into individual pots containing vermiculite and soil substrate (1:2 v/v) and maintained at 24:20 °C day:night and a photoperiod of 16:8 h light:dark until their use.

As a model NCT virus, the viral isolate M06 of CMV, kindly provided by Dr. E. Moriones (IHSM-La Mayora-CSIC, Málaga, Spain), was used. CMV single- and double-infected melon plants were mechanically inoculated at phenological stage 12 (BBCH scale) with fresh macerated material from infected plants. Melon plants were used 4 weeks post-inoculation.

As a model circulative virus, we used an isolate of CABYV, kindly provided by Dr. H. Lecoq, obtained from zucchini squash from Montafavet, France, in 2003, and conserved in the laboratory by serial transmission through the aphid vector *A. gossypii*. Briefly, and as described in Moreno et al. (2011) with some modifications, to obtain CABYV single- and double-infected plants, apterous adult aphids of *A. gossypii* were allowed to feed for an acquisition access period of 72 h on CABYV single-infected plants. After this period, 20 CABYV-viruliferous aphids were placed inside clip cages (1 per plant) on melon plants at stage 12 (BBCH scale) for an inoculation access period (IAP) of 48 h on the test plants. After this IAP, aphids and nymphs were removed from all plants, leaving them completely clean. Test plants were checked daily to ensure no aphids or nymphs were present on infected plants.

To obtain CABYV/CMV double-infected melon plants, CMV was mechanically inoculated 72 h after CABYV inoculation by viruliferous aphids, following the procedure described above and using different leaves to inoculate each virus (Galán-Cubero et al. 2025). Infected plants were checked for infection by visual inspection, Enzyme-Linked Immunosorbent Assay (ELISA) (Adams and Clark 1977) and RT-qPCR. Infected plants were used as virus source plants 4 weeks post-inoculation.

### Virus Detection and Accumulation Analysis

Virus infection by CMV and CABYV in single- and double-infected melon plants was determined by Double and Triple Antibody Sandwich ELISA, respectively, using specific antibodies following the standard protocol provided by the manufacturers (Bioreba and DSMZ, respectively). CMV and CABYV infection was also verified by visual inspection. CMV and CABYV viral titers in infected melon plants were estimated by RT-qPCR (Galán-Cubero et al. 2025). Briefly, 25× RT-PCR Enzyme Mix and 2× RT-PCR Buffer (Applied Biosystems) were combined with: TaqMan probe (CMV-M6-MGB 5′-CCGACGTATGATTGTCCT-3′ and CABYV TaqMan-MGB 3976 [Moreno et al. 2011] probes final concentration 3 μM); forward and reverse primers (CMV_MP_2F 5′-CCCGCTTTGGTGTCTTTCC-3′/CMV_MP_1R 5′-CCGCTTACGATTCCCAACTG-3′ and CABYV_3956.F/CABYV_4013.R [Moreno et al. 2011] final concentration of 10 μM); 50 ng DNA; and nuclease-free water for a final reaction volume of 25 μl. Cycling parameters were as follows: 45 °C for 10 min and 95 °C for 15 min; 40 cycles of 95 °C for 15 s and 55 °C for 15 s. Each sample was tested in triplicate, and the absolute number of copies in the samples quantified using the standard curve made from amplified DNA fragments of CMV and CABYV using the primers previously described. It was constructed from dilutions of CMV and CABYV transcripts obtained using the MEGAscript transcription kit (ThermoFisher) following the manufacturer’s instructions. The equation used for the standard curve of CABYV was *y* = −3.2354x + 36.489; and that of CMV was *y* = −3.2616x + 41.443. Plants with similar virus accumulation were selected for use in assays.

### Life-History Traits of the Aphid *A. gossypii* on CMV and CABYV Single- and Double-Infected Plants

The impact of CABYV and CMV, as single and double infections, on the life history of the vector *A. gossypii* was evaluated using the protocol described by Fereres et al. (1989) as follows. Four treatments were compared: (i) CMV single-infected plants; (ii) CABYV single-infected plants; (iii) CABYV/CMV double-infected plants; and (iv) mock-inoculated plants used as controls.

Two adult aphids of the same age, taken from the synchronized aphid colony, were placed, using a clip cage, on a leaf with clear virus symptoms of each replicate melon plant (stage 17, BBCH scale) in each of the 4 treatments. Thus, aphids were placed on the oldest leaves of CABYV single-infected plants and on the youngest expanded leaves of CMV single-infected plants. For CABYV/CMV double-infected plants, intermediate leaves showing symptoms of both viruses were selected. Intermediate leaves were also used for mock-inoculated plants. After 24 h, both the adults and nymphs, except 1, were removed from each clip cage. Once these nymphs reached adulthood, the number of progeny they produced was counted daily (and then removed) for a period equivalent to the prereproductive period (d).

Population parameters evaluated were the intrinsic rate of natural increase [*r_m_* = 0.738 (ln*Md*)/*d*] and prereproductive period (*d*) (Wyatt and White 1977); the mean relative growth rate [*RGR* = (0.86 × ln*Md*)/*d*]; mean generation time (*Td* = *d*/0.738); effective fecundity (offspring produced during a time interval equivalent to the prereproductive period, *Md*); and adult aphids were weighed at the end of the assay (on the day of the last count). Replicates in which the aphid died before determining its weight were not considered in the analysis. There were 6 to 18 replicate plants (and thus aphids) per treatment; the entire experiment was repeated on 3 occasions under the same experimental conditions (Table 1). Briefly, 8, 9, and 8 plants were used as mock-inoculated plants during the first, second, and third occasions the experiment was repeated, respectively. In the case of the CMV single-infected treatment, 13, 11, and 6 plants were used for the first, second, and third occasions the experiment was repeated, respectively. For CABYV single-infected treatment, 6, 16, and 12 plants were used for the first, second, and third occasions the experiment was repeated, respectively. Lastly, in the treatment using CMV/CABYV double-infected plants, 8, 18, and 9 plants were used for the first, second, and third occasions the experiment was repeated, respectively. Differences in the number of replicates used during each repetition were due to the success in generating single and double infections, in addition to aphid survival when placed on the test plants.

**Table 1.** Population parameters of *Aphis gossypii* on CMV and CABYV single- and double-infected plants

Variables	Virus treatments								Statistical analysis	
	Mock		CMV		CABYV		CABYV/CMV		ANOVA	Kruskal–Wallis
	***n***	Mean ± SE	***n***	Mean ± SE	***n***	Mean ± SE	***n***	Mean ± SE		
**Weight**	21	0.559 ± 0.033 a	30	0.423 ± 0.020 b	32	0.449 ± 0.019 b	33	0.409 ± 0.024 b	*F* = 6.802, *P* < 0.001	
**Td**	25	9.756 ± 0.207 b	30	9.666 ± 0.169 b	34	9.844 ± 0.175 b	35	9.795 ± 0.167 b		*H* = 0.688, *P* = 0.876
**d**	25	7.200 ± 0.153 b	30	7.130 ± 0.124 b	34	7.260 ± 0.129 b	35	7.230 ± 0.124 b		*H* = 0.688, *P* = 0.876
**RGR**	25	0.457 ± 0.008 b	30	0.419 ± 0.007 a	34	0.458 ± 0.006 b	35	0.452 ± 0.011 b		*H* = 19.180, *P* < 0.001
**Md**	25	44.800 ± 1.279 b	30	32.670 ± 1.638 a	34	47.470 ± 1.519 b	35	46.600 ± 2.269 b	*F* = 14.679, *P* < 0.001	
***r*_m_**	25	0.393 ± 0.007 b	30	0.360 ± 0.006 a	34	0.394 ± 0.005 b	35	0.389 ± 0.010 b		*H* = 19.180, *P* < 0.001

Results of the weight (mg), mean relative growth rate (*RGR*); mean generation time (*Td*, days); effective fecundity (*Md*, nymphs); intrinsic rate of natural increase (*r_m_*), and prereproductive period (*d*, days) in the 3 replicates tested. Different letters indicate significant differences (*P* < 0.05) according to ANOVA (Gaussian variables) or Kruskal–Wallis (non-Gaussian variables).

### Alighting and Settling Behavior of *A. gossypii* on CABYV/CMV Double-Infected Plants

Two different greenhouse experiments were done to assess alighting and settling preferences of nonviruliferous alate *A. gossypii* on mock-inoculated or CABYV/CMV double-infected melon plants at serial time intervals after aphid release. Greenhouse conditions consisted of a photoperiod of 16:8 h light:dark at 25:20 °C day:night. Alighting and settling experiments were conducted following the methodology of Carmo-Sousa et al. (2014, 2016) with some modifications. Alate (winged) morphs of *A. gossypii* were used. To obtain them, aphids from our colony were allowed to complete 3 generations (21 to 23 days) in the same cages with the same plants, so the population became overcrowded, which is known to enhance production of alate morphs. The alate colony was maintained under greenhouse conditions with a photoperiod of 16:8 h light:dark at 25:20 °C day:night.

The effect of CMV and CABYV as single infections on the alighting and settling *A. gossypii* have already been addressed by Carmo-Sousa et al. (2014, 2016) under the same experimental conditions we used here; consequently, they were not included in this study.

#### Alighting Behavior

**Fig. 1.**
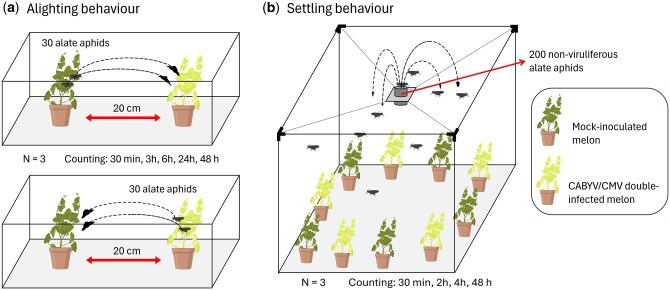
Diagram of experimental setup to investigate alighting and settling behavior of *A. gossypii* on CABYV/CMV double-infected plants. Both experiments were repeated on 3 occasions. (a) Thirty *A. gossypii* alate aphids were released either on a mock-inoculated or a CABYV/CMV double-infected melon plant depending on the treatment and allowed to settle on the plant of release or migrate to the other plant. Aphids were then counted 30 min, 3 h, 6 h, 24 h, and 48 h after release using the same cage for the different evaluation periods. (b) Two-hundred *A. gossypii* alate aphids were released in a cage where they could choose between settle on mock-inoculated or CABYV/CMV double-infected melon plants. Aphids were counted 30 min, 2 h, 4 h, and 48 h after release using the same cage for the different evaluation periods.

One CABYV/CMV double-infected and 1 mock-inoculated melon plant were placed 20 cm apart inside a transparent aphid-proof cage with uniform lighting. The position of the infected and mock-inoculated plants was alternated between replicates to avoid any possible effects of external factors such as light or temperature on the behavior of the aphids. To evaluate the plant-mediated indirect effect of CMV/CABYV double infection on alighting behavior, groups of 30 nonviruliferous alates were collected with a manual aspirator from the colony and the group released onto a leaf of either the CABYV/CMV double-infected plant or the mock-inoculated plant, depending on the experimental treatment (in each cage, aphids were initially placed on the infected or the healthy plant) (Fig. 1). For each replicate (cage), the number of alates remaining on the plant onto which they had been introduced and the number that had moved and successfully settled on the other plant in the cage were recorded visually, and counts were repeated on the same plants after 30 min, 3 h, 6 h, 24 h, and 48 h using a mirror to avoid touching the plant and interfering with the natural behavior of the insects because the same cage was used for the different time intervals. In total, there were 15 replicates for each treatment that were performed over 3 occasions (5 replicates per occasion). A new set of alate aphids was used for each replicate. The settling rate for each treatment and time interval was calculated as the mean percentage of aphids that migrated from the plant onto which they had been released and successfully settled on the other plant in the cage.

#### Settlement Behavior

The preference of nonviruliferous *A. gossypii* alates to settle on either CABYV/CMV double-infected or mock-inoculated melon plants was tested at different time intervals under choice conditions according to the protocol previously described by Carmo-Sousa et al. (2014, 2016) with some modifications. Briefly, a total of 10 test plants, 5 CABYV/CMV double-infected and 5 mock-inoculated plants, were placed alternately in a circle inside a 1 m^3^ cage equipped with an aphid-proof mesh net. As controls, to check there was no interference with external factors, there were 2 additional control cages; 1 had 10 mock-inoculated plants and the other had 10 CABYV/CMV double-infected plants. Two hundred nonviruliferous alate aphids were placed on a flight platform as described by Fereres et al. (1999) located at the top of each cage and 0.5 m above the plant canopy. Aphids were then released into the cage from the flight platform so they could land and settle on any of the test plants (Fig. 1). The experiment was repeated 3 times for each evaluation period. The number of alate aphids settled on each test plant (either on the mock-inoculated or on the CABYV/CMV double-infected melon plants) was counted after a short time (30 min, 2 h, and 4 h) and a long time (48 h) interval using a mirror to avoid touching the plants and consequently modifying the insect behavior. Moreover, nymphs were also counted at 48 h. Each mean value was obtained from 15 plants per treatment (5 plants per treatment and per cage × 3 replicated cages).

### Statistical Analysis

All parameters evaluated were analyzed using SPSS version 29.0.0.0 (IBM Corp. 2024) and checked for normality using the Shapiro–Wilk *W*-test. In the life-history trait experiments, variables that did not follow a normal distribution were transformed using sqrt (*x* + 1) and ln (*x* + 1). After transformation, a parametric ANOVA was used to compare treatments with Gaussian variation, followed by a Fisher’s least significant difference (LSD) test for comparisons between treatment means. Where treatment variation was non-Gaussian, comparisons were made with the nonparametric Kruskal–Wallis *H*-test, followed by post-hoc LSD tests with Bonferroni adjustment.

Regarding alighting experiments, the percentage of aphids that migrated to the opposite plant to which they were released in each treatment was transformed by arcsin[sqrt(x)] before analysis. Comparisons between treatments were made using a Mann–Whitney *U*-test since variables followed a non-Gaussian distribution. For aphid settlement, the mean number of alate aphids settled was analyzed using a linear mixed-effects model fitted by restricted maximum likelihood. The fixed effects included treatment, time, and their interaction (treatment × time), using type III sums of squares. The cage was included as a random effect with an identity covariance structure to account for random variability among replicates. Repeated measurements over time within each experimental unit (the plant is treated as a pseudoreplicate) were modeled using a heterogeneous compound symmetry covariance structure. Individual comparisons of the number of alate aphids settled and the total number of nymphs between treatments (mock-inoculated versus CABYV/CMV double-infected melon plants) at each time interval were performed using a Student *t*-test. Statistical significance was assessed at the 0.05 level.

## Results

### Life-History Traits of the Aphid *A. gossypii* on CMV and CABYV Single- and Double-Infected Plants

To determine the effect of CMV and CABYV single- and double-infected melon plants on fitness of the aphid *A. gossypii*, life-history assays were performed.

There was no significant difference in mean generation time (Td) or prereproductive period (d) among treatments (Td: *H* = 0.688, *P* = 0.876; d: *H* = 0.688, *P* = 0.876). However, certain parameters varied significantly when comparing results from CMV single-infected plants with those from other treatments. There were significantly fewer nymphs (Md) on plants infected only with CMV (32.670 ± 1.638 nymphs) compared with plants that received other treatments (mock-inoculated: 44.800 ± 1.279 nymphs; CABYV: 47.470 ± 1.519 nymphs; CABYV/CMV: 46.460 ± 2.269 nymphs) (*P* < 0.05). There was also no significant difference in Md between mock-inoculated plants and those infected with CABYV in single or CABYV/CMV double infection (*P* > 0.05). As a consequence, both mean RGR and intrinsic rate of population increase (*r_m_*) were also lower for aphids maintained on CMV single-infected plants (RGR: 0.419 ± 0.007; *r_m_*: 0.360 ± 0.006) than on other treatments (RGR: 0.457 ± 0.008 and *r_m_*: 0.393 ± 0.007 for mock-inoculated plants; RGR: 0.458 ± 0.006 and *r_m_*: 0.394 ± 0.005 for CABYV single-infected plants; and RGR: 0.452 ± 0.011 and *r_m_*: 0.389 ± 0.010 for CABYV/CMV double-infected plants) (*P* < 0.05); there was no significant difference between the other 3 treatments (mock-inoculated, CABYV single- and CABYV/CMV double-infected melon plants) (*P* > 0.05) (Table 1).

Furthermore, aphids that fed on mock-inoculated plants were significantly heavier (0.559 ± 0.033 mg) than those fed on infected plants, regardless of the type of virus infection (CMV: 0.423 ± 0.020 mg; CABYV: 0.449 ± 0.019 mg; CABYV/CMV: 0.409 ± 0.024 mg) (*P* < 0.05). There were no significant differences in weight among virus-infected treatments (*P* > 0.05) (Table 1).

### Alighting and Settling Behavior of *A. gossypii* on CABYV/CMV Double-Infected Plants

#### Alighting Behavior

**Fig. 2.**
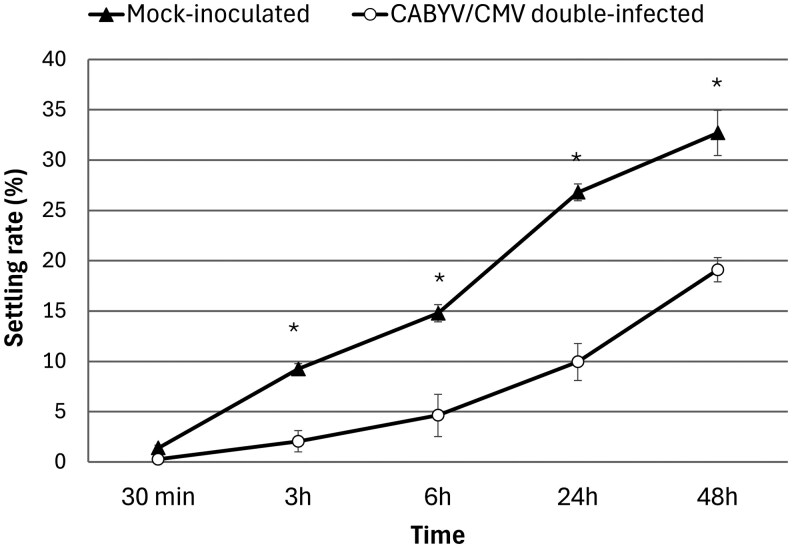
Effects of CABYV/CMV double-infected melon plants on the behavior of *A. gossypii*. The proportion (%) of *A. gossypii* alates that migrated from a CABYV/CMV double-infected plant to a mock-inoculated one (black triangle) and vice versa (white circle) 30 min, 3 h, 6 h, 24 h, and 48 h after aphid introduction. *Significant differences according to Mann–Whitney *U*-test (non-Gaussian variables) (*P* < 0.05). Error bars represent the SE.

There was no significant difference in aphid host plant preference between mock-inoculated (1.406% ± 0.533% aphids) and CABYV/CMV double-infected melon plants (0.278% ± 0.278% aphids) after a 30-min interval (*U* = 83.000, *P* = 0.084), although more aphids tended to settle on mock-inoculated than on double-infected plants (5× more). There was a clear shift after medium and longer time intervals (3, 6, 24, and 48 h) in aphids migrating from CABYV/CMV double-infected to mock-inoculated melon plants (Fig. 2). Thus, at medium time intervals, more aphids were found on mock-inoculated (3 h: 9.258% ± 1.066% aphids; 6 h: 14.787% ± 2.100% aphids) than on double-infected plants (3 h: 2.064% ± 0.517% aphids; 6 h: 4.647% ± 0.843% aphids) (3 h: *U* = 0.000, *P* < 0.001; 6 h: *U* = 23.000, *P* < 0.001). Similar results were observed after long time intervals; more aphids were found on mock-inoculated (24 h: 26.802% ± 1.840% of aphids; 48 h: 32.705% ± 1.200% of aphids) than CABYV/CMV double-infected plants (24 h: 9.956% ± 0.851% of aphids; 48 h: 19.103% ± 2.244% of aphids) (24 h: *U* = 0.000, *P* < 0.001; 48 h: *U* = 21.000, *P* < 0.001) (Fig. 2).

#### Settling Behavior

Type III tests of fixed effects revealed no significant main effect of treatment (*F* = 3.26, *P* = 0.082), whereas time had a significant effect on the measured variable (*F* = 5.30, *P* = 0.003). Importantly, the interaction between treatment and time was highly significant (*F* = 26.51, *P* < 0.001), indicating that the effect of treatment varied across the different time points.

**Fig. 3.**
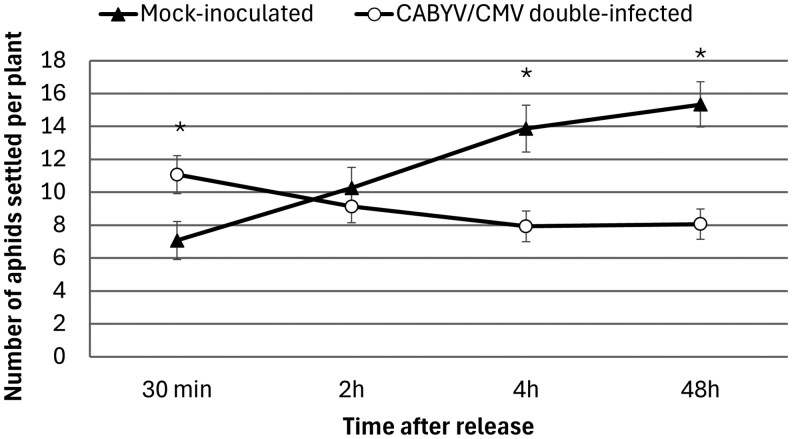
Number (mean ± SE) of *A. gossypii* alates present on CABYV/CMV double-infected (white circles) and mock-inoculated melon plants (black triangles) 30 min, 2 h, 4 h, and 48 h after aphid release. *Significant differences according to Student *t*-test (*P* < 0.05). Error bars represent the SE.

When comparisons of the number of settled alate aphids between treatments were performed over time, the results showed that *A. gossypii* alates significantly preferred CABYV/CMV double-infected (11.067 ± 1.152 aphids) melon plants over mock-inoculated melon plants (7.067 ± 1.152 aphids) after 30 min (*P* < 0.05). However, at the 2 h interval, there was no significant difference in the number of aphids settled on CABYV/CMV double-infected (9.133 ± 0.995 aphids) and mock-inoculated plants (10.267 ± 1.248 aphids) (*P* = 0.484). After longer periods of time, aphids preferred to settle on mock-inoculated (4 h: 13.867 ± 1.420 aphids; 48 h: 15.333 ± 1.369 aphids) rather than on CABYV/CMV double-infected plants (4 h: 7.933 ± 0.933 aphids; 48 h: 8.067 ± 0.923 aphids) (*P* < 0.05), suggesting that aphids rejected to feed on infected plants after longer evaluation periods (Fig. 3).

**Fig. 4.**
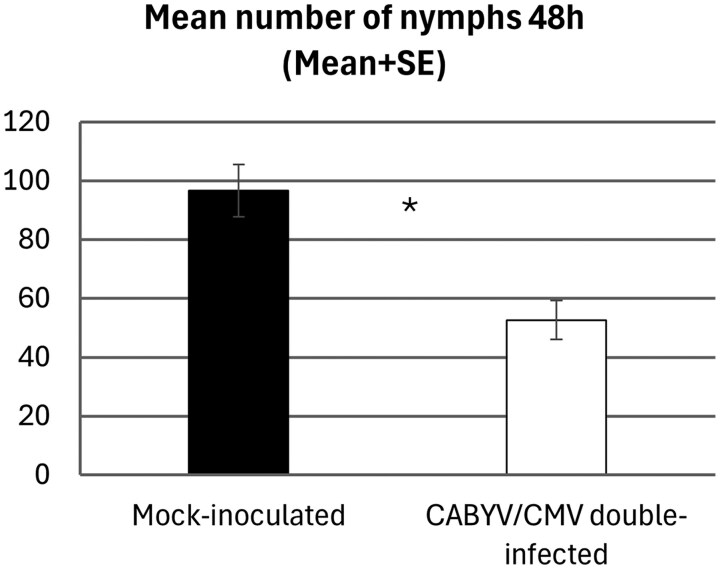
Number (mean ± SE) of *A. gossypii* nymphs present on CABYV/CMV double-infected and mock-inoculated melon plants 48 h after release of alate adults under free-choice conditions. *Significant differences according to Student *t*-test (*P* < 0.05). Error bars represent the SE.

Results for the mean number of nymphs 48 h after release reflected the results obtained for host plant preference of alates, which preferred mock-inoculated plants compared with double-infected plants after the same period. The total number of nymphs was much higher (almost double) on mock-inoculated (96.600 ± 8.834 nymphs) plants than on CABYV/CMV double-infected melon plants (52.667 ± 6.633 nymphs) (*t* = 0.416, d.f. = 28, *P* < 0.001) (Fig. 4).

## Discussion

Mixed virus infections are very common in the field and important in plant virus ecology and epidemiology (Moreno and López-Moya 2020). Over the course of infection, some viruses exert direct and indirect effects on their vectors, altering their behavior, physiology, and performance to increase their own dispersal (Mauck et al. 2010, 2014, Bak et al. 2017). Our study aimed to explore the significance of mixed infections (CABYV and CMV) on the fitness and host plant preference of the cotton aphid, *A. gossypii*, on melon plants.

Our work demonstrated that virus infection had a significant influence on the fitness of *A. gossypii*, particularly when aphids remain on CMV single-infected melon plants. The reduced intrinsic rate of increase, effective fecundity, and RGR observed for aphids feeding on CMV single-infected melon plants indicated poor adaptability to this host, which reduced its population growth, thereby reinforcing the hypothesis that virus–plant–vector interactions modulate aphid development and reproduction. These findings are consistent with previous studies on NCT viruses, which have reported negative effects on vector fitness. For instance, studies done by Mauck et al. (2010) showed reduced population growth of *A. gossypii* and *M. persicae* when feeding on CMV single-infected zucchini plants. Moreover, *Aphis glycines* growth on soybean plants infected with another NCT virus, alfalfa mosaic virus (AMV, *Alfamovirus*), was almost 20% lower than that for aphids developing on uninfected plants (Donaldson and Gratton 2007). Similar patterns in aphid reproduction were observed with *A. pisum* feeding on bean plants infected with the NC *Potyvirus* BYMV (Hodge and Powell 2008). This negative trend in population growth has also been observed in other insect vectors, such as *B. tabaci*, which laid 6 times more eggs on mock-inoculated watermelon plants than on watermelon infected with squash vein yellowing virus (*Potyviridae*) (Shrestha et al. 2017). Conversely, TuMV-infected *Arabidopsis thaliana* and *N. benthamiana* plants attracted *M. persicae* and promoted their reproduction compared with mock-inoculated plants (Casteel et al. 2014). These beneficial effects on the vector align with observations of CMV-infected pepper plants, on which *M. persicae* increased reproduction compared with mock-inoculated plants (Warnasooriya et al. 2024). These contrasting results suggest that virus effects on their insect vectors depend on the specific virus–vector–host plant combination, because CMV reduces *A. gossypii* fitness on melon but can also enhance *M. persicae* reproduction on pepper. This outcome could result from an alteration in plant metabolites or plant defense responses that modulate vector fitness, highlighting the complexity of pathosystem interactions where virus-induced changes in plant traits can have varying effects depending on the host plant species. Another explanation is that viruses cannot be perfectly adapted to manipulate every host species they infect, a concept highlighted in recent reviews on virus–vector–plant coevolution (García-Arenal and Fraile 2013, Lefeuvre et al. 2019, Mauck and Chesnais 2020, Chesnais et al. 2025).

Conversely, CABYV infection did not significantly impact aphid fitness, exhibiting a neutral effect similar to that reported in other persistently transmitted viruses, such as PEMV in *A. pisum*, where no changes in aphid survival, growth, or reproduction were observed (Hodge and Powell 2008). Interestingly, some CT viruses, such as BYDV in *S. avenae*, PLRV in *M. persicae*, and Bean leafroll virus (BLRV) in *A. pisum*, enhance vector development, increasing population growth rates (Fereres et al. 1989, Castle and Berger 1993, Davis et al. 2017). Similar effects were observed in another CT virus; the growth rate of *M. persicae* was enhanced when feeding on TuYV single-infected *Camelina sativa* plants (Chesnais et al. 2019). Thus, our results suggest that, if the “Vector Manipulation Hypothesis” is correct, CABYV would not necessarily require enhanced vector fitness to improve its own spread; alternatively, CABYV may simply not be adapted to manipulate all host species to increase vector fitness. Additional factors that could influence virus spread include emission of volatiles by virus-infected plants and yellowing of infected tissues, making them more attractive to aphids favoring aphid prealighting behavior and settlement on infected host plants (Fereres and Moreno 2009, Bosque-Pérez and Eigenbrode 2011).

Interestingly, no significant differences were observed in aphid biological parameters between CABYV/CMV double-infected plants and mock-inoculated plants, suggesting a compensatory effect between CMV and CABYV. Indeed, CMV alone reduced aphid fitness while CABYV maintained a neutral effect, but their combination may counterbalance the negative effect of CMV, resulting in an overall neutral outcome. Similar results were obtained by Peñaflor et al. (2016), where double infection with bean pod mottle virus (*Comoviridae*) and soybean mosaic virus (SMV, *Potyvirus*) eliminated the negative effects of nonpersistent SMV single infection on *A. glycines* population growth. Lightle and Lee (2014) also reported no significant differences in the prereproductive development time of *Amphorophora agathonica* aphids feeding on uninfected raspberry plants and those double infected with raspberry leaf mottle virus (*Closterovirus*) and raspberry latent virus (*Reovirus*). This supports the hypothesis that mixed infections could modulate vector responses, potentially influencing virus transmission dynamics.

The alighting and settling experiments revealed that nonviruliferous *A. gossypii* alates initially preferred CABYV/CMV double-infected plants over mock-inoculated plants. However, as time progressed (2 h and beyond), this host plant preference diminished, and aphids that had been feeding on infected plants (and most likely had become viruliferous) significantly preferred to move and settle on uninfected plants after 4 h. Our findings expand those reported by Carmo-Sousa et al. (2014), who observed that nonviruliferous *A. gossypii* alates were initially attracted to CMV single-infected plants, but after some time, they preferred to settle on mock-inoculated cucumber plants. The preference of CABYV viruliferous *A. gossypii* alates for mock-inoculated cucumber plants from the early stages of their assays was similar to results we obtained using double-infected plants (Carmo-Sousa et al. 2016). This behavioral modification underlines the role of aphid movement during virus epidemiology, where initial attraction to infected sources may enhance virus acquisition, while subsequent dispersal increases the likelihood of secondary infections (Mauck et al. 2010). Moreover, Carmo-Sousa et al. (2016) reported that nonviruliferous *A. gossypii* showed no preference for mock-inoculated or CABYV single-infected cucumber plants, suggesting that CABYV does not drive aphid attraction in this pathosystem. This may explain why, in our study, aphids were first drawn to CABYV/CMV double-infected plants, influenced by CMV, but later, once they presumably became viruliferous, migrated toward uninfected plants as a result of the combined effects of both viruses. The epidemiological implications of these virus-induced changes in aphid behavior tend to promote virus spread because while CMV predominantly governs aphid attraction and initial settlement under our experimental conditions, the overall plant physiological changes induced by double infection ultimately promote aphid dispersal and virus acquisition, thereby facilitating the spread of both viruses in the field.

In addition to attraction driven by visual, olfactory, and gustatory cues, feeding behavior during mixed infections is shaped by plant infection status and is related not only to virus transmission but also to host plant recognition during the early stages of plant selection. For instance, Galán-Cubero et al. (2025) reported that *A. gossypii* showed increased intracellular puncturing of epidermal and mesophyll cells and longer phloem sap ingestion when feeding on CABYV/CMV double-infected melon plants. This enhanced probing activity on superficial tissues promotes the acquisition of CMV before aphids abandon infected plants in search of more suitable hosts. Moreover, it alters the aphids’ status, making them viruliferous after they ingest phloem sap, particularly important for CT viruses, followed by migration to uninfected hosts, further facilitating the transmission of viruses such as CABYV (Mauck 2016).

On the other hand, the significant reduction in the number of nymphs found on CABYV/CMV double-infected plants compared with mock-inoculated plants is consistent with the findings of Carmo-Sousa et al. (2014, 2016), who found more nymphs on mock-inoculated plants than CMV or CABYV single-infected cucumber plants. However, our life-history trait experiment under controlled conditions did not show a decrease in aphid fitness on CABYV/CMV double-infected plants. Thus, the higher number of nymphs found on uninfected plants could be attributed to a greater settlement rate of winged aphids on these plants. This suggests that, under greenhouse conditions, the lower incidence of nymphs on CABYV/CMV double-infected plants does not necessarily indicate reduced aphid fitness but may instead be influenced by environmental factors or differences in the alighting and settlement behavior of winged aphids.

Our combined results on aphid host plant preference and fitness highlight how mixed infections can alter vector behavior and virus transmission in different ways. While CMV single infections negatively affected *A. gossypii* fitness, this expected effect of CMV on aphid fitness was not observed in plants with double infections, indicating that CABYV counteracts the detrimental impact of CMV on aphids. This neutral fitness outcome may help retain aphids on infected plants temporarily, which could increase the likelihood of CABYV acquisition, as prolonged feeding is required for efficient uptake of circulative viruses. Thus, emigration of aphids from CABYV/CMV double-infected plants to uninfected plants may be due to visual or olfactory cues coming from symptomatic CABYV/CMV double-infected plants. These changes in aphid behavior could be attributed to virus-induced alterations in plant physiology that might affect phloem composition, volatile composition, or secondary metabolites, thereby facilitating aphid attraction and settlement (Eigenbrode et al. 2002, Mauck et al. 2010, Carmo-Sousa et al. 2014, 2016). These changes could be due to virus infection within the host plant and render infected plants less preferred to aphids after initial feeding and might explain the significantly higher weight of aphids feeding on uninfected plants compared with those feeding on infected ones, suggesting superior nutritional quality (Villalba et al. 2024). Additionally, Galán-Cubero et al. (2025) observed that the duration of phloem sap ingestion was significantly shorter on uninfected plants than on CMV and CABYV single- and double-infected plants, suggesting that aphids would require less feeding ingestion time on uninfected hosts due to their superior nutritional quality. This mechanism aligns with the observed reduction in aphid fitness on CMV single-infected plants, because the negative impact on aphid growth and reproduction may drive them to abandon these hosts in search of more suitable ones, such as uninfected plants. Importantly, despite the presence of CABYV in double infection, it did not prevent the aphids’ eventual departure from CABYV/CMV double-infected melon plants, further increasing virus dispersal efficiency. This suggests that the effects of CMV on host plant quality and aphid behavior may outweigh any potential stabilizing influence of CABYV. Consequently, since CABYV relies on prolonged phloem feeding for efficient transmission, early attraction of aphids to CABYV/CMV double-infected melon plants, followed by aphid dispersal to mock-inoculated plants, could reduce the time available for persistent virus acquisition. This pattern may limit the spread of CABYV compared with CMV, which benefits from the aphid’s initial attraction and subsequent migration to uninfected plants, optimizing its nonpersistent transmission strategy.

Overall, these findings highlight how mixed infections can modify aphid behavior in ways that promote virus dispersal, as described by other authors (Mauck et al. 2010, Carmo-Sousa et al. 2014, Liang et al. 2025). By initially attracting aphids but later discouraging long-term settlement, CABYV/CMV double-infection creates a scenario in which, once they become viruliferous, aphids are more likely to spread CABYV and CMV to uninfected plants. The contrasting effects of CMV and CABYV on *A. gossypii* fitness highlight the complexity of mixed infections and their implications for virus epidemiology. Further research should explore the underlying molecular and biochemical mechanisms driving these interactions and responses, particularly under field conditions, where additional environmental factors could modulate aphid behavior and virus transmission dynamics. Taken together, our results highlight the fundamental role of mixed infections in the ecology of plant viruses and underscore the need for further research to better understand the underlying mechanisms regulating these dynamics. A deeper understanding of these dynamics will improve predictions of virus spread and support the development of more effective disease management strategies.
