# Correction to: Consequences of double virus infections on host plant preference and performance of *Aphis gossypii* (Hemiptera: Aphididae)

**DOI:** 10.1093/aesa/saag023

**Published:** 2026-05-31

This is a correction to: Rocio Galan-Cubero, Javier Cazorla, Alberto Fereres, Aranzazu Moreno, Consequences of double virus infections on host plant preference and performance of *Aphis gossypii* (Hemiptera: Aphididae), *Annals of the Entomological Society of America*, 2026; saag009, https://doi.org/10.1093/aesa/saag009

The originally-published version of this manuscript omitted to designate authors Alberto Fereres and Aranzazu Moreno as co-corresponding authors. This error has been corrected. 

